# Magnetic resonance imaging and ultrasound fusion technique in gynecology

**DOI:** 10.1002/uog.24754

**Published:** 2022-01-12

**Authors:** M. Bazot, F. Spagnoli, S. Guerriero

**Affiliations:** ^1^ Department of Radiology, Tenon University Hospital, Assistance Publique des Hôpitaux de Paris (AP‐HP) Sorbonne University Paris France; ^2^ Groupe de Recherche Clinique (GRC‐6), Centre Expert en Endométriose (C3E), Assistance Publique des Hôpitaux de Paris Tenon University Hospital, Sorbonne University Paris France; ^3^ GE Healthcare University Panthéon Sorbonne (Paris I) Paris France; ^4^ Centro Integrato di Procreazione Medicalmente Assistita (PMA) e Diagnostica Ostetrico‐Ginecologica, University of Cagliari Policlinico Universitario Duilio Casula Monserrato Cagliari Italy

## Introduction

The development of imaging methods over the last 50 years has revolutionized medicine, broadening the diagnostic possibilities in a non‐invasive way. The use and storage of digital data allow immediate review of information obtained. More recently, the coupling of imaging techniques has developed through fusion methods. These combine the advantages of two individual imaging methods (e.g. ultrasound, computed tomography (CT), magnetic resonance imaging (MRI), fluorodeoxyglucose‐positron emission tomography (^19^FDG‐PET)) by superimposing their diagnostic information.

In the past decade, fusion of MRI and ultrasound data has grown in popularity, in particular in intestinal and prostate imaging^1^, ^2^. In addition to its diagnostic contribution, this technique of fusion allows optimization of biopsy sampling by facilitating a targeted approach^3^, ^4^. However, the MRI‐ultrasound fusion technique is underdeveloped in the field of obstetrics and gynecology. Preliminary studies have suggested its potential in identifying deep pelvic endometriosis and Cesarean section scars^5^, ^6^, and more recent studies have also underlined the potential value of fusion of CT or ^19^FDG‐PET with ultrasound in the evaluation of gynecological cancers^7^, ^8^, ^9^, ^10^. In order to determine the potential contribution of MRI‐ultrasound fusion, since 2019, we have undertaken to apply it to 160 patients with suspected endometriosis selected to undergo joint investigation by MRI and transvaginal ultrasound.

The MRI‐ultrasound fusion technique involves coupling of the images, achieved by means of an electromagnetic support and sensors placed on the ultrasound probe. The sensors enable the position of the probe to be identified within a space, allowing synchronization of ultrasound with MRI acquisitions previously loaded into the ultrasound system. Automatic or manual synchronization of MRI and ultrasound images can be used, ensuring that a given point visualized on ultrasound imaging corresponds to the same point on the MR image.

In order to optimize the MRI‐ultrasound fusion technique, there are a number of technical considerations that need to be addressed, concerning conventional ultrasound imaging and MRI protocols, the MRI and ultrasound fusion imaging technique, indications for the fusion technique in gynecological imaging and the limitations of ultrasound and MRI.

## Conventional ultrasound imaging and MRI protocols

**Figure 1:**
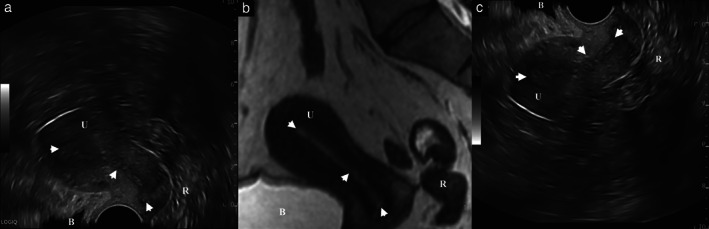
(a,b) Simultaneous display of corresponding sagittal planes obtained by transvaginal sonography (TVS) (a) and magnetic resonance imaging (MRI) (b), illustrating different landmarks (arrowheads) used to synchronize the TVS and MRI techniques for fusion imaging, and the main structures analyzed (bladder (B), uterus (U) and rectum (R)). The bladder may be filled differently in the MRI acquisition and on real‐time ultrasound, which can impair the quality of the examination. (c) Additional reconstructed top‐down TVS image.

Transvaginal ultrasound is the first‐line examination globally for any gynecological pathology. Yet, despite its universal application, there is no international consensus on the image orientation of the standard reference plane^11^. Sagittal analysis of a female pelvis can be performed by the sonographer according to either of two potential imaging planes: top‐down (‘gynecology plane’) or bottom‐up (‘radiology plane’). Hence, gynecologists and radiologists often work with images that are the ‘inverse’ of each other's. For both specialties, looking at the acquisition screen, in the midsagittal view, the bladder is located on the left and the rectum on the right, the transducer being at the top in gynecology (top‐down) and at the bottom in radiology (bottom‐up) (Figure 1). In a recent study, Taksøe‐Vester *et al*.^11^ demonstrated that bottom‐up compared with top‐down image orientation on transvaginal ultrasound imaging led to steeper learning curves, with fewer attempts and less time needed to reach expert levels of performance.

There is, however, an international consensus on the orientation of MR images. A midsagittal acquisition displays anatomically, from left to right, the pubic symphysis, bladder and urethra, uterus and vagina, rectum and anal canal, and sacrum and coccyx. The same elements are visualized similarly during a bottom‐up sagittal transvaginal ultrasound acquisition (except the bony structures are less well visualized). It therefore seems logical to adopt this common orientation to perform transvaginal ultrasound examinations, particularly those intended for ultrasound‐MRI fusion (Figure 1).

## MRI and ultrasound fusion imaging

### Acquisition and storage of data

Due to the limited number of published studies, there is as yet no consensus on the optimal requirements for fusion of ultrasound and MRI. This technique is probably best achieved with acquisition of both modalities on the same day, thereby optimizing synchronization of the pelvic organs. The fusion technique can be performed by a radiologist alone or by a gynecologist ultrasound examiner assisted by a radiologist. The MRI examination should always be done first, in order to allow image transfer from the Picture Archiving Communication System (PACS), or, failing that, from a CD ROM (recordable compact disc), to the ultrasound machine. In our practice, MR images are acquired at 1.5 Tesla (T), using a Signa® HDxT (GE Healthcare, Milwaukee, WI, USA) scanner, or at 3T, using a Signa™ Architect (GE Healthcare) scanner, and ultrasound images are acquired with a LOGIQ E10 (GE Healthcare) machine.

Certain prerequisites concerning the MRI protocol have been defined by a recent European consensus conference^12^. Optimizing MRI quality involves the application of simple principles, including fasting for 3 hours prior to the scan, administering a digestive/colon preparation by enema the day before or at least a few hours prior to the study, and having the patient empty their bladder before commencement of each imaging modality. Subcutaneous or intravenous injection of an antiperistaltic agent (e.g. Glucagen®) is highly recommended before any MRI examination to limit bowel‐movement artifacts. After MRI, care must be taken to ensure that there are no episodes of hypoglycemia (post‐Glucagen injection) that may interfere with performance of the secondary ultrasound‐MRI fusion imaging. Using ultrasound gel for vaginal or rectal opacification is not recommended, because this can modify the natural anatomical relationships.

**Figure 2:**
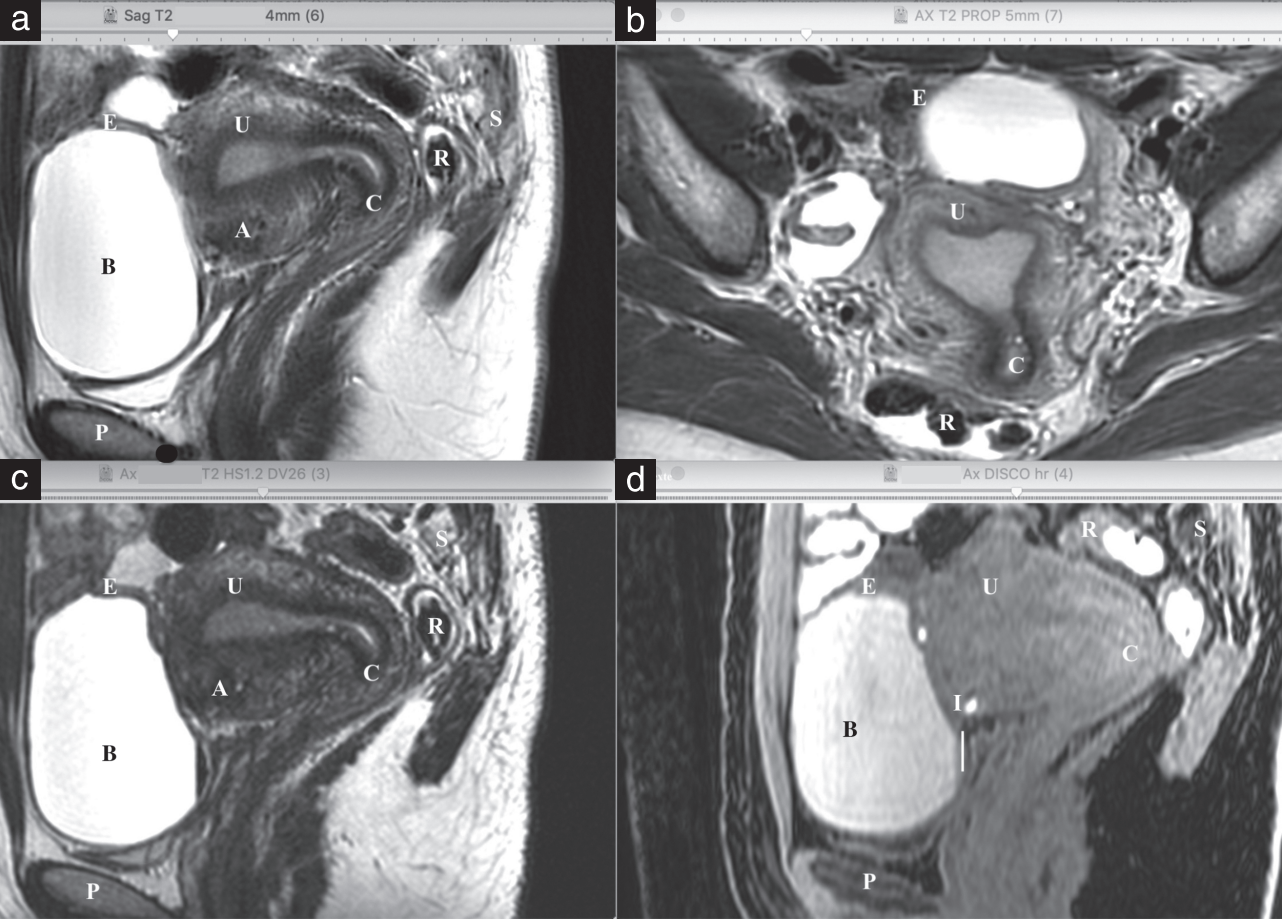
Sagittal two‐dimensional (2D) T2‐weighted (T2) fast‐spin‐echo (FSE) (a), axial 2DT2 FSE (b), reformatted sagittal three‐dimensional (3D) T2 (c) and 3D T1‐weighted with fat‐suppression (d) magnetic resonance imaging sequences in the same patient, displaying the different pelvic structures and organs: pubic symphysis (P), bladder (B), uterus (U), cervix (C), rectosigmoid colon (R) and sacrum (S). Note presence of endometrial cyst (E), superficial peritoneal implants (I) and external adenomyosis (uterine endometriosis) (A).

**Figure 3:**
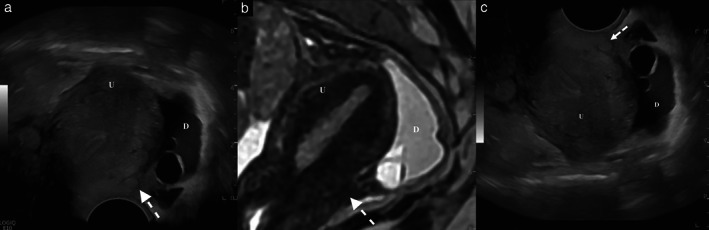
Fusion imaging in a patient with superficial peritoneal and retrocervical endometriotic lesions. Corresponding sagittal planes obtained by transvaginal sonography (TVS) (a) and magnetic resonance imaging (MRI) (b), with additional reconstructed top‐down TVS image (c), showing the uterus (U) and the pouch of Douglas (D). In both modalities, the presence of pelvic fluid containing cystic hemorrhagic peritoneal lesions and deep retrocervical endometriosis can be seen, which is echogenic on TVS and hypointense on T2‐weighted MRI (arrows).

A conventional MRI protocol uses at least sagittal and axial two‐dimensional (2D) T2‐weighted (T2) fast‐spin‐echo MRI sequences, regardless of the suspected pathology (Figure 2). Therefore, initially, these two sequences were integrated in the gynecological imaging examinations carried out prior to performing the fusion technique. This approach, however, is not optimal, due to the technological constraints of ultrasound acquisition. Transvaginal ultrasound provides sagittal and coronal views, but, unlike MRI, it is unable to provide an axial view directly. This technological limitation of transvaginal ultrasound accentuates the value of three‐dimensional (3D) T2 and 3D T1‐weighted (T1) Dixon sequences (Figure 2). These 3DT2 and 3DT1 volume acquisitions are isotropic, allowing infinite multiplanar slices that are perfectly suited to fusion imaging. In addition to natural spontaneous contrast resolution, MRI has very good spatial resolution, allowing optimal correlation with ultrasound data. The use of a 3DT2 acquisition volume allows the sonographer to overcome limitations of analysis plane (Videoclip S1). The addition of a 3DT1 Dixon acquisition allows correlation of ultrasound with T1 contrast (phase imaging) and with fat‐suppression (water imaging), optimizing, for example, the search for small hemorrhagic endometriotic implants (Figure 3). There is no recommendation regarding the use of gadolinium in the evaluation of deep pelvic endometriosis in the European Society of Urogenital Radiology (ESUR) guidelines^12^. Hence, post‐contrast 3DT1 MRI cannot be recommended for the ultrasound‐MRI fusion technique. To summarize, for the fusion of ultrasound with MRI, the use of 3DT2 and 3DT1 Dixon MRI sequences is preferred.

### Synchronization of MRI and ultrasound images

The success of the MRI‐ultrasound fusion technique relies on the precision of image synchronization, so that the same structure examined by MRI and ultrasound has the same spatial coordinates for each modality. This is achieved quite easily when studying a stationary organ with fixed anatomy and small size, such as the prostate^3^. Overall examination of the female pelvis, however, poses more of a challenge in terms of image acquisition, which can be automatic or manual. The placement of three spatial landmarks on the patient's abdominal wall during the acquisition of MRI sequences should allow automatic synchronization by the ultrasound machine. So far, however, a lack of recognized landmarks has prevented this method from being adopted, and synchronization is carried out manually, by placing three successive points (e.g. uterine fundus, isthmus, exocervix or urethral orifice) on the ultrasound image and then on the MR image (Figure 1). This essential step represents one of the major current limitations of ultrasound‐MRI fusion in gynecology (Videoclip S2); a recent preliminary study of 10 patients with suspected endometriosis showed a significant lack of synchronization of the ultrasound and MR images^13^.

Of note, use of the term ‘fusion’ in this situation is in fact somewhat inappropriate, because this method involves side‐by‐side analysis of successive sectional planes. True fusion of ultrasound and MRI acquisitions is possible, but it requires very precise adjustment of the two modalities (Videoclip S3).

## Indications for fusion imaging in gynecology

There is currently no recognized indication for fusion imaging in gynecology. However, pelvic endometriosis is a field in which its application may be of particular interest. Preliminary studies have demonstrated its feasibility and current limitations^5^, ^13^. Coupling MRI with ultrasound could make it possible to optimize the detection of superficial peritoneal (hemorrhagic implants) and ovarian (deep implants and differential diagnoses between endometriotic cysts and hemorrhagic luteal) lesions and to improve the detection of deep endometriotic locations (Figure 3). A recent Cochrane review reported the diagnostic accuracy of ultrasound and MRI for deep endometriosis: only rectosigmoid involvement was evaluated with similar accuracy by the two imaging techniques, while, for other deep pelvic locations, the diagnostic accuracy of ultrasound and MRI differed considerably^14^. In this setting, the coupling of these modalities could optimize detection of deep pelvic endometriotic lesions, particularly at the most common locations, i.e. the uterosacral ligaments.

A preliminary study has shown that the MRI‐ultrasound fusion technique can detect adenomyosis, but the absence of a histological gold standard limits the applicability of this approach^15^. Leiomyomas/fibroids and adenomyosis represent the two most common uterine pathologies, and these are frequently associated with each other. The contribution of fusion imaging coupled with power Doppler should enable a differential diagnosis between leiomyoma and adenomyoma.

Several recent studies have highlighted the potential value of fusion imaging for some gynecological cancers^7^, ^9^, ^10^, ^16^. These studies suggested that fusion of MRI or CT and ultrasound is feasible in patients with locally advanced cervical or ovarian cancer and may increase the diagnostic accuracy relative to that of the individual imaging methods used alone^10^, ^16^.

Another study showed that 3D single‐photon‐emission computed tomography/computed tomography images from prior cross‐sectional examinations could be coupled with visualization in real time of lymph‐node architecture by linear array or transvaginal ultrasound, and that this may help to identify the target lymph node, guiding the examiner to perform a core‐needle biopsy or to inject radiotracer for selective surgical nodal excision, using a radio‐guided occult lesion localization technique^7^.

## Limitations of ultrasound

Transvaginal ultrasound is the first‐line investigation for exploring the female pelvis. It is a simple, accessible, reproducible and inexpensive modality, characterized by high spatial resolution. However, it is important to highlight two technological limitations, of geometric and mathematical origin, that are specific to ultrasound. Unlike MRI, ultrasound has limitations in terms of contrast resolution, limiting tissue characterization of pelvic structures. Ultrasound vocabulary has always referred erroneously to sagittal, transverse and oblique reference acquisition planes. This terminology is inaccurate, since transvaginal ultrasound provides sagittal, coronal and oblique acquisitions. Only a suprapubic pelvic ultrasound examination can produce strict axial views of the female pelvis. However, the benefits of suprapubic ultrasound do not compensate for its limitations in terms of spatial resolution, and transvaginal analysis remains the best option. The problem of the inability of transvaginal ultrasound to provide a strict axial view could possibly be compensated for by acquisition of a 3D ultrasound volume, but, in the context of fusion imaging, this is not possible currently, as the probes dedicated to ultrasound‐MRI fusion are not volumetric.

The second technological limitation is related to the power of the ultrasound beam, and the nature and depth of the anatomical structures studied, which are themselves affected by the patient's morphology. In addition, the presence of locally advanced disease (endometriosis or malignancy) can distort the anatomy, making it impossible to use reproducible planes during ultrasound imaging. To ensure the best image quality, the probe should be either inserted deeply or almost withdrawn from the vagina and rotated to different angles until the region of interest is visualized clearly. There is another potential problem related to movement on ultrasound, especially in the assessment of endometriosis or locally advanced cervical cancer. A dynamic aspect of the ultrasound examination combines the sliding sign, to assess the mobility of the pelvic organs, with tenderness‐guided ultrasound, in which the organs are moved with the transvaginal ultrasound probe. This aids in mapping endometriosis or pelvic adhesions. However, this means that, during ultrasound, organs may be moved relative to their position on the MR images. Similarly, in cervical cancer, to assess tumor growth in surrounding organs (bladder or rectum), the mobility (or adhesion) of organs relative to each other is evaluated, which improves the diagnostic accuracy of ultrasound (in terms of negative predictive value) but prohibits the fusion technique.

## Limitations of MRI

MRI is the second‐line investigation for exploring the female pelvis. It is a moderately accessible and expensive modality, characterized by high‐contrast resolution. Although a few studies showed 3DT2 to be lower in quality than the other sequences, 3DT2 MRI acquisition is highly recommended for the ultrasound‐MRI fusion technique^17^, ^18^, ^19^, ^20^. MRI provides better tissue characterization than does transvaginal ultrasound, but the use of three different weightings (T2, T1 and T1 with fat‐suppression) is required.

## Conclusion

The MRI‐ultrasound fusion technique is a new, non‐invasive diagnostic tool that shows considerable potential in gynecological practice. Combining these two modalities may improve the detection of abnormalities beyond that possible using either modality alone. Prospective studies dedicated to specific gynecologic diseases, such as endometriosis, are required to standardize this new tool and assess its value.

## Supporting information


**Videoclip S1** 3D T2‐weighted magnetic resonance imaging sequence showing multiple anatomical continuous planes (coronal, axial and sagittal) and corresponding views of different normal pelvic organs (bladder, ovaries with follicles and rectosigmoid) and abnormalities (superficial uterine adenomyosis with multiple high‐signal‐intensity tiny cysts within normal junctional zone on low signal intensity, Nabothian cysts within endocervical canal, and small amount of fluid in the pouch of Douglas).

**Videoclip S2** Ultrasound‐magnetic resonance imaging (MRI) fusion showing different sagittal and coronal oblique planes obtained by transvaginal sonography and MRI analyzed side‐by‐side. Note that a perfect match is not obtained for some pelvic structures (e.g. Nabothian cyst, ovaries).

**Videoclip S3** Ultrasound‐magnetic resonance imaging (MRI) fusion using a real fusion technique modality, in which ultrasound and MRI analysis are merged together. This technique appears suboptimal for dedicated analysis of pelvic organs and structures.

## Data Availability

Data available on request from the authors.
